# Rifaximin Attenuates Liver Fibrosis and Hepatocarcinogenesis in a Rat MASH Model by Suppressing the Gut–Liver Axis and Epiregulin–IL-8-Associated Angiogenesis

**DOI:** 10.3390/ijms26146710

**Published:** 2025-07-12

**Authors:** Naoki Nishimura, Kosuke Kaji, Norihisa Nishimura, Junichi Hanatani, Tatsuya Nakatani, Masafumi Oyama, Akihiko Shibamoto, Yuki Tsuji, Koh Kitagawa, Shinya Sato, Tadashi Namisaki, Satoru Tamaoki, Hitoshi Yoshiji

**Affiliations:** 1 Department of Gastroenterology, Nara Medical University, Kashihara 634-8521, Nara, Japan; 2 Medical Affairs Department, ASKA Pharmaceutical Co., Ltd., Minato-ku 108-8532, Tokyo, Japan

**Keywords:** liver cirrhosis, hepatocellular carcinoma, metabolic dysfunction-associated steatohepatitis, gut-liver axis

## Abstract

Metabolic dysfunction-associated steatohepatitis (MASH) is a progressive liver disease linked to fibrosis and hepatocellular carcinoma (HCC). Gut-derived lipopolysaccharide (LPS) promotes hepatic inflammation, fibrosis, and angiogenesis through toll-like receptor 4 (TLR4) signaling. This study examined the effects of rifaximin, a non-absorbable, gut-targeted antibiotic, on MASH-related liver fibrosis and early hepatocarcinogenesis, with a focus on the LPS–epiregulin–IL-8–angiogenesis axis.MASH was induced in Fischer 344 rats using a choline-deficient, L-amino acid-defined high-fat diet (CDAHFD). Rifaximin (30 mg/kg/day) was orally administered for 12 weeks. Liver histology, gene expression, intestinal permeability, LPS levels, and angiogenic markers were evaluated. Rifaximin reduced hepatic inflammation, fibrosis, hydroxyproline content, and fibrogenic gene expression. The number and size of GST-P-positive preneoplastic lesions and proliferation-related genes were decreased. Portal LPS levels and Kupffer cell activation declined, with downregulation of *Lbp*, *Cd14*, *Tlr4*, and inflammatory cytokines. Rifaximin decreased hepatic epiregulin and IL-8 expression, attenuated CD34-positive neovascularization, and suppressed proangiogenic gene expression, accompanied by improved intestinal barrier function and reduced gut permeability. Rifaximin mitigates MASH progression by restoring gut barrier integrity, limiting LPS translocation, and inhibiting fibrogenic and angiogenic pathways. These results suggest its potential as a chemopreventive agent in MASH-related hepatocarcinogenesis.

## 1. Introduction

Metabolic dysfunction-associated steatohepatitis (MASH), formerly known as nonalcoholic steatohepatitis (NASH), is a progressive form of fatty liver disease associated with metabolic syndrome. MASH has become a leading cause of chronic liver disease worldwide, paralleling the global rise in obesity and type 2 diabetes [1,2,3,4,5]. MASH is characterized by hepatic steatosis, inflammation, and varying degrees of fibrosis, which can progress to cirrhosis and hepatocellular carcinoma (HCC) [1,2,3,4,5]. Liver fibrosis is the primary determinant of liver-related morbidity and mortality in MASH, since advanced fibrosis significantly increases the risk of HCC [6]. Despite the growing awareness of its clinical impact, limited pharmacological therapies have been approved for halting or reversing fibrosis in MASH. Lifestyle modifications remain the cornerstone of treatment, but due to limited adherence, there remains an urgent need for effective and targeted therapies. The mechanisms linking fibrogenesis to hepatocarcinogenesis in MASH involve a complex interplay of chronic inflammation, oxidative stress, genetic alterations, and cellular senescence [7,8,9]. However, these are not yet completely understood, and this gap in mechanistic insight hampers the development of preventive strategies for HCC. Therefore, these challenges must be addressed to mitigate the rising burden of MASH and its long-term complications [10].

Gut-derived lipopolysaccharide (LPS), which is translocated into the portal circulation due to intestinal dysbiosis and barrier dysfunction, has been increasingly recognized as a key contributor toward hepatocarcinogenesis [11,12,13,14,15]. In the liver, LPS activates toll-like receptor 4 (TLR4) found in Kupffer cells, which initiates various proinflammatory signaling cascades [16,17]. This leads to a sustained production of cytokines such as TNF-α and IL-6, fostering a chronic inflammatory milieu that promotes hepatocyte proliferation, DNA damage, and malignant transformation [16,17]. Additionally, LPS-TLR4 signaling induces hepatic stellate cell (HSC) activation and fibrogenesis, further contributing toward a tumor-promoting microenvironment [18,19]. LPS also enhances hepatic angiogenesis, a hallmark of tumor progression, through the upregulation of proangiogenic factors [20,21]. In response to LPS, activated macrophages and endothelial cells secrete vascular endothelial growth factor, which promotes neovascularization that supports tumor growth and metastasis [20]. This LPS-driven angiogenesis facilitates vascular remodeling and increased microvessel density, which are commonly observed in HCC [18,20,21]. This positions gut-derived LPS as a potential therapeutic target for attenuating fibrosis and suppressing hepatocarcinogenesis in MASH.

Rifaximin is a gut-selective antibiotic that inhibits bacterial RNA synthesis, reduces gut-derived toxins, and modulates the microbiota. Although it is commonly used for treating hepatic encephalopathy for chronic liver diseases, it has also demonstrated potential benefits in MASH by altering the gut microbiota composition, reducing intestinal permeability, and lowering circulating LPS [22,23,24,25]. In preclinical studies of MASH models, rifaximin attenuated liver steatosis, inflammation, and fibrosis through suppression of TLR4-mediated signaling pathways [23,24,25]. However, it remains unclear whether these effects of rifaximin on the gut–liver axis (specifically angiogenesis) can inhibit hepatic carcinogenesis.

In the hopes of identifying a novel chemopreventive agent, this study investigated the effect of rifaximin on liver fibrosis and carcinogenesis in a MASH rat model, focusing on changes in angiogenesis associated with intestinal-derived LPS regulation.

## 2. Results

### 2.1. Rifaximin Attenuates Steatohepatitis in CDAHFD-Fed Rats

**Figure 1:**
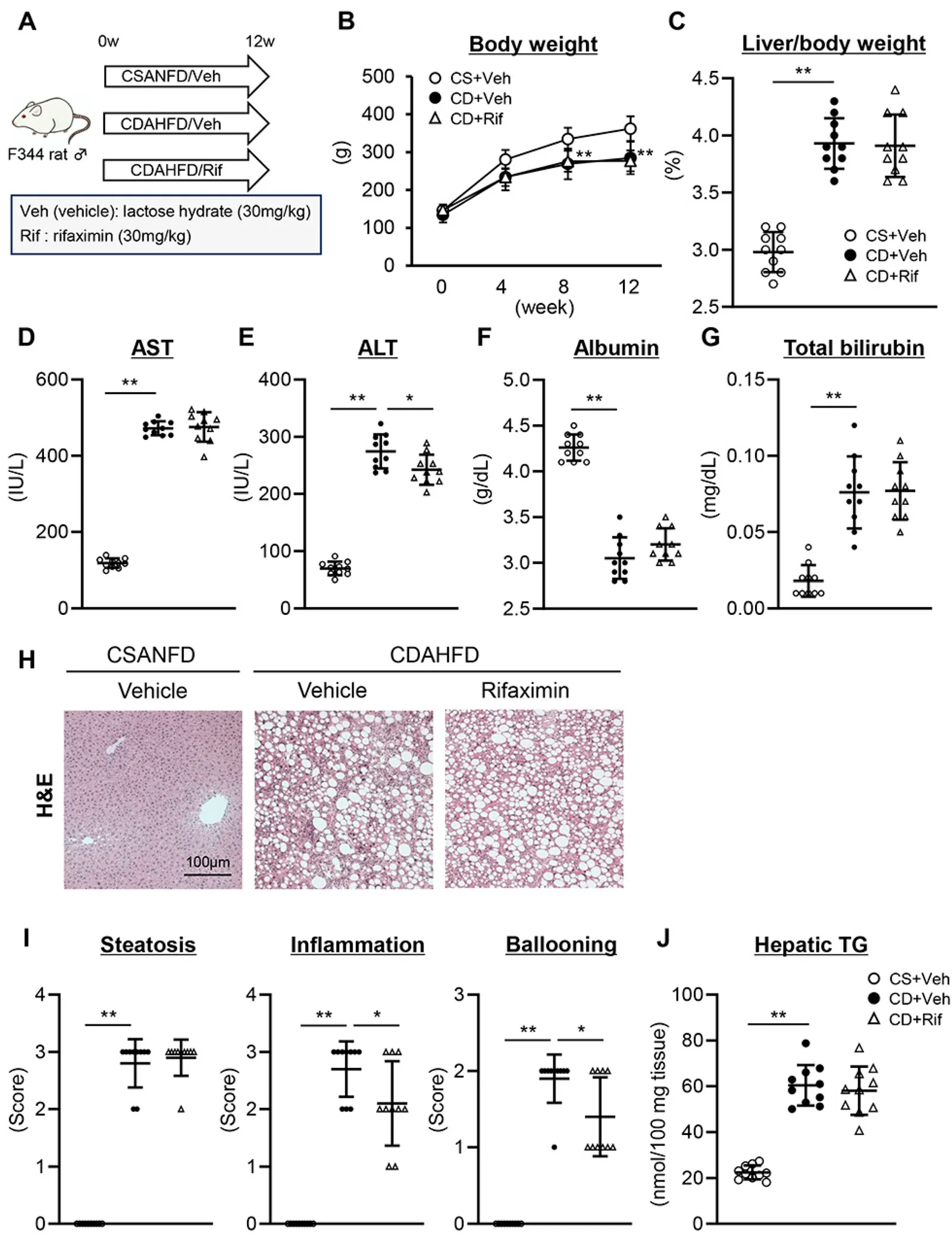
Effect of rifaximin on CDAHFD-induced steatohepatitis. (**A**) Schematic of rifaximin administration to CDAHFD-fed rats. (**B**) Body weight during the experimental period. (**C**) Ratio of liver to body weight at the end of the experiment. (**D**–**G**) Serum levels of (**D**) AST, (**E**) ALT, (**F**) albumin, and (**G**) total bilirubin at the end of the experiment. (**H**) Representative microphotographs of hematoxylin and eosin (H&E) staining of liver tissue. (**I**) NAS (Non-alcoholic Steatohepatitis Activity Score) based on H&E staining. (**J**) Hepatic triglyceride (TG) level. Data are the mean ± SD (*n* = 10). ** *p* < 0.01 vs. CS+Veh group at each time point (**B**), * *p* < 0.05, ** *p* < 0.01, significant difference between groups determined by Student’s *t*-test (**C**–**G**,**I**,**J**).

The effect of rifaximin on CDAHFD-induced steatohepatitis was examined (Figure 1A). The CDAHFD-fed rats demonstrated marked weight loss compared to the CSANFD-fed rats, and the administration of rifaximin did not affect the body weight of the CDAHFD-fed rats (Figure 1B). Similarly, CDAHFD-fed rats exhibited hepatomegaly, which was unaffected by rifaximin administration (Figure 1C). CDAHFD-fed rats also demonstrated an increase in serum aspartate transaminase (AST), alanine aminotransferase (ALT), and bilirubin, as well as a decrease in albumin, but only the increase in serum ALT was attenuated by rifaximin (Figure 1D–G). On histological assessment, the CDAHFD-fed rats had severe steatohepatitis characterized by steatosis, inflammation, and hepatocyte ballooning, among which rifaximin attenuated only the latter two (Figure 1H,I). Consistently, rifaximin did not affect the hepatic TG content in CDAHFD-fed rats (Figure 1J). 

### 2.2. Rifaximin Suppresses Liver Fibrosis and Hepatocarcinogenesis in CDAHFD-Fed Rats

**Figure 2:**
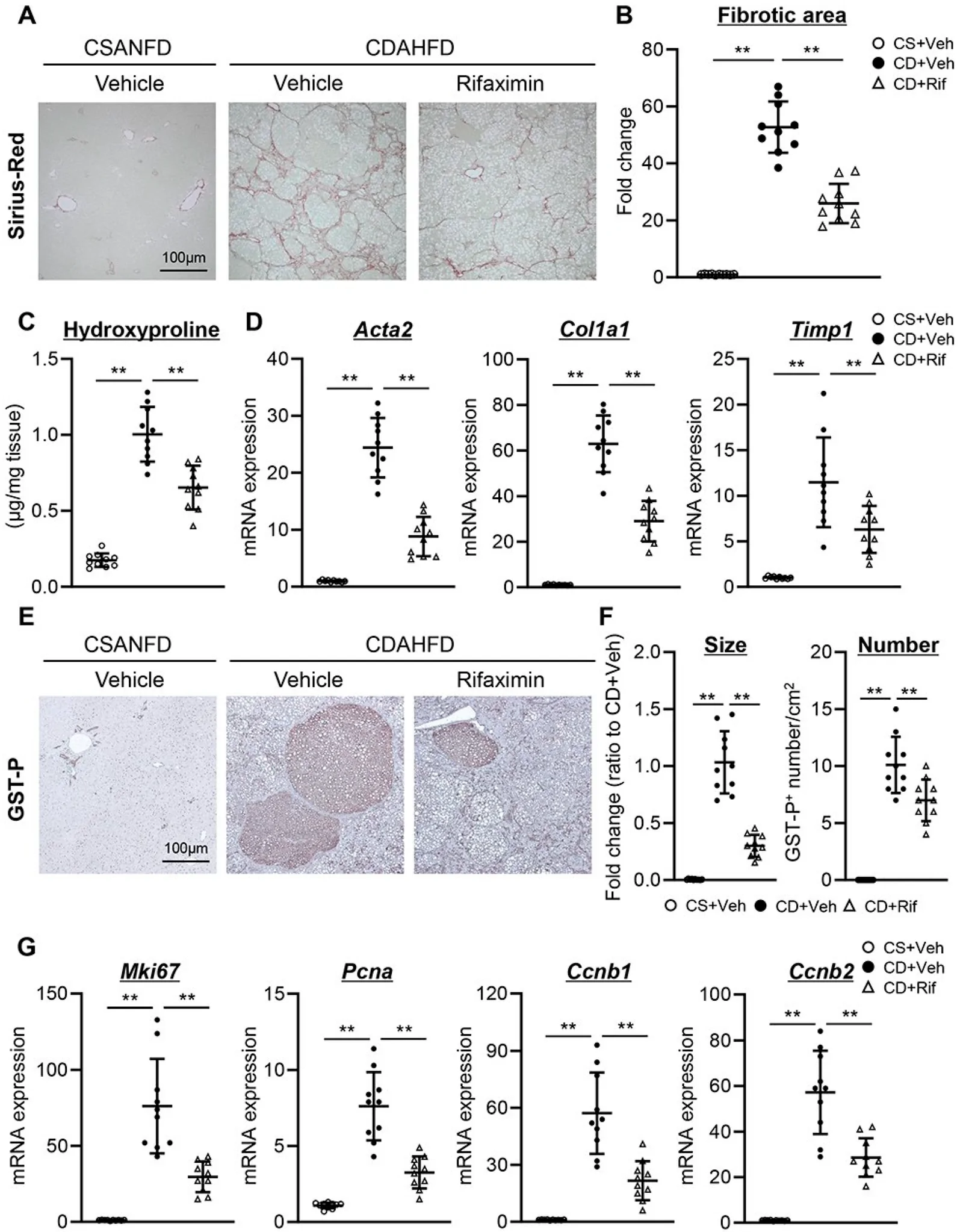
Effect of rifaximin on CDAHFD-induced liver fibrosis and hepatocarcinogenesis. (**A**) Representative microphotographs of Sirius-Red staining of liver tissue. (**B**) Quantification of Sirius-Red stained fibrotic area in high-power field. (**C**) Hepatic concentration of hydroxyproline. (**D**) Hepatic mRNA level of profibrotic markers (*Acta2*, *Col1a1*, and *Timp1*). (**E**) Representative microphotographs of placental glutathione transferase (GST-P)-positive preneoplastic foci in the experimental rats. (**F**) Number and relative size of GST-P-positive neoplastic lesions per square centimeter. (**G**) Hepatic mRNA level of genes related to cell proliferation and cell cycle (*Mki67*, *Pcna*, *Ccnb1*, and *Ccnb2*). *Gapdh* was used as an internal control for qRT-PCR (**D**,**G**). Data are the mean ± SD (*n* = 10). Quantitative values are indicated as fold changes to the values of the CS+Veh group (**B**,**D**,**G**) or the CD+Veh group (**F**). ** *p* < 0.01, significant difference between groups determined by Student’s *t*-test.

The CDAHFD-fed rats developed extensive liver fibrosis, which was suppressed by rifaximin to about 50% (Figure 2A,B). Rifaximin also decreased the concentration of hydroxyproline in the liver tissue (Figure 2C), as well as the hepatic mRNA levels of profibrogenic genes (*Acta2*, *Col1a1*, and *Timp1*) (Figure 2D). Next, to determine the effect of rifaximin on hepatocarcinogenesis, semi-quantitative analyses were used to assess the number and size of GST-P-positive preneoplastic lesions (GST-P^+^ PNL) in the liver tissues; these were significantly reduced after treatment with rifaximin (Figure 2E,F). Additionally, rifaximin also attenuated the upregulation of *Mki67*, *Pcna*, *Ccnb1*, and *Ccnb2*, which are key regulators of cell proliferation and cell cycle progression in hepatocarcinogenesis (Figure 2G).

### 2.3. Rifaximin Reduces Portal LPS Levels and Ameliorates Hepatic LPS/TLR4-Mediated Kupffer Cell Activation

**Figure 3:**
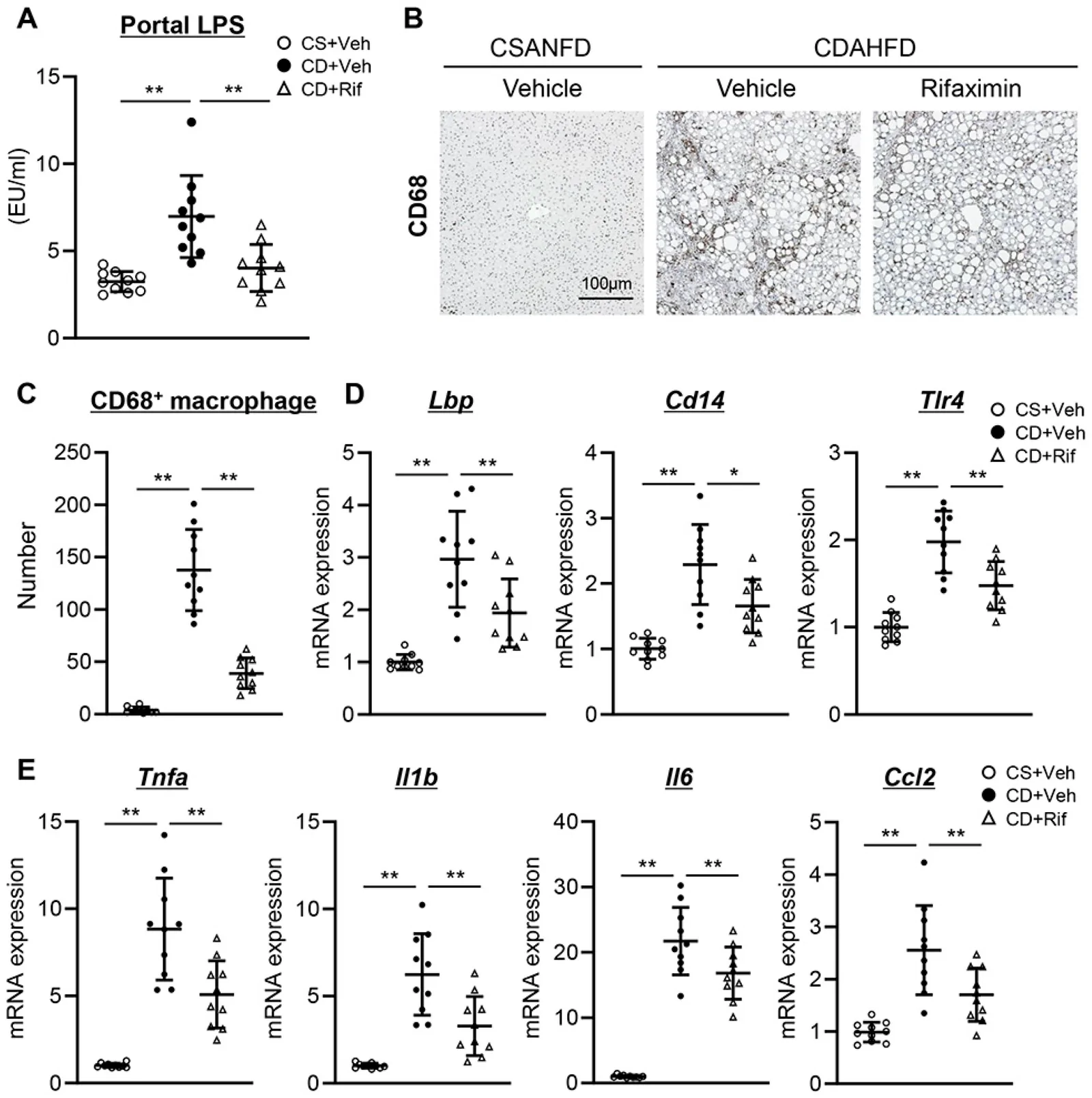
Effect of rifaximin on LPS/TLR4 signaling in the liver of CDAHFD-fed rats. (**A**) Serum LPS level in the portal vein. (**B**) Representative microphotographs of CD68 staining of liver tissue. (**C**) Quantification of CD68-stained macrophages in a high-power field. (**D**) Hepatic mRNA level of genes related to LPS-TLR4 signaling (*Lbp*, *Cd14*, and *Tlr4*). (**E**) Hepatic mRNA level of proinflammatory cytokine genes (*Tnfa*, *Il1b*, *Il6*, and *Ccl2*). *Gapdh* was used as an internal control for qRT-PCR (**D**,**E**). Data are the mean ± SD (*n* = 10). Quantitative values are indicated as fold changes to the values of the CS+Veh group (**D**,**E**). * *p* < 0.05, ** *p* < 0.01, significant difference between groups determined by Student’s *t*-test.

Since gut-derived LPS is known to contribute to liver fibrosis and hepatocarcinogenesis, we evaluated the impact of rifaximin on the influx of LPS into the liver and LPS-induced Kupffer cell activation [11,12,13,14,15,16,17,18,19,20,21]. Portal LPS levels were increased in the CDAHFD-fed rats compared to CSANFD-fed rats, and this was attenuated by treatment with rifaximin (Figure 3A). In line with this, the expansion of CD68-positive Kupffer cells in CDAHFD-fed rats was also suppressed by rifaximin (Figure 3B,C). LPS-binding protein (LBP) binds LPS and transfers it to CD14, which presents it to TLR4, thereby initiating inflammatory signaling in Kupffer cells. In addition to reducing portal LPS levels and suppressing Kupffer cell activation, treatment with rifaximin reduced the hepatic expression of *Lbp*, *Cd14*, and *Tlr4* (Figure 3D). As a result of the aforementioned LPS/TLR4-dependent suppression of Kupffer cell activation, rifaximin significantly suppressed the hepatic expression of proinflammatory cytokines (*Tnfa*, *Il1b*, *Il6*, and *Ccl2*) in CDAHFD-fed rats as well (Figure 3E).

### 2.4. Rifaximin Reduces Epiregulin and Inhibits IL-8-Associated Neovascularization in the Liver of CDAHFD-Fed Rats

**Figure 4:**
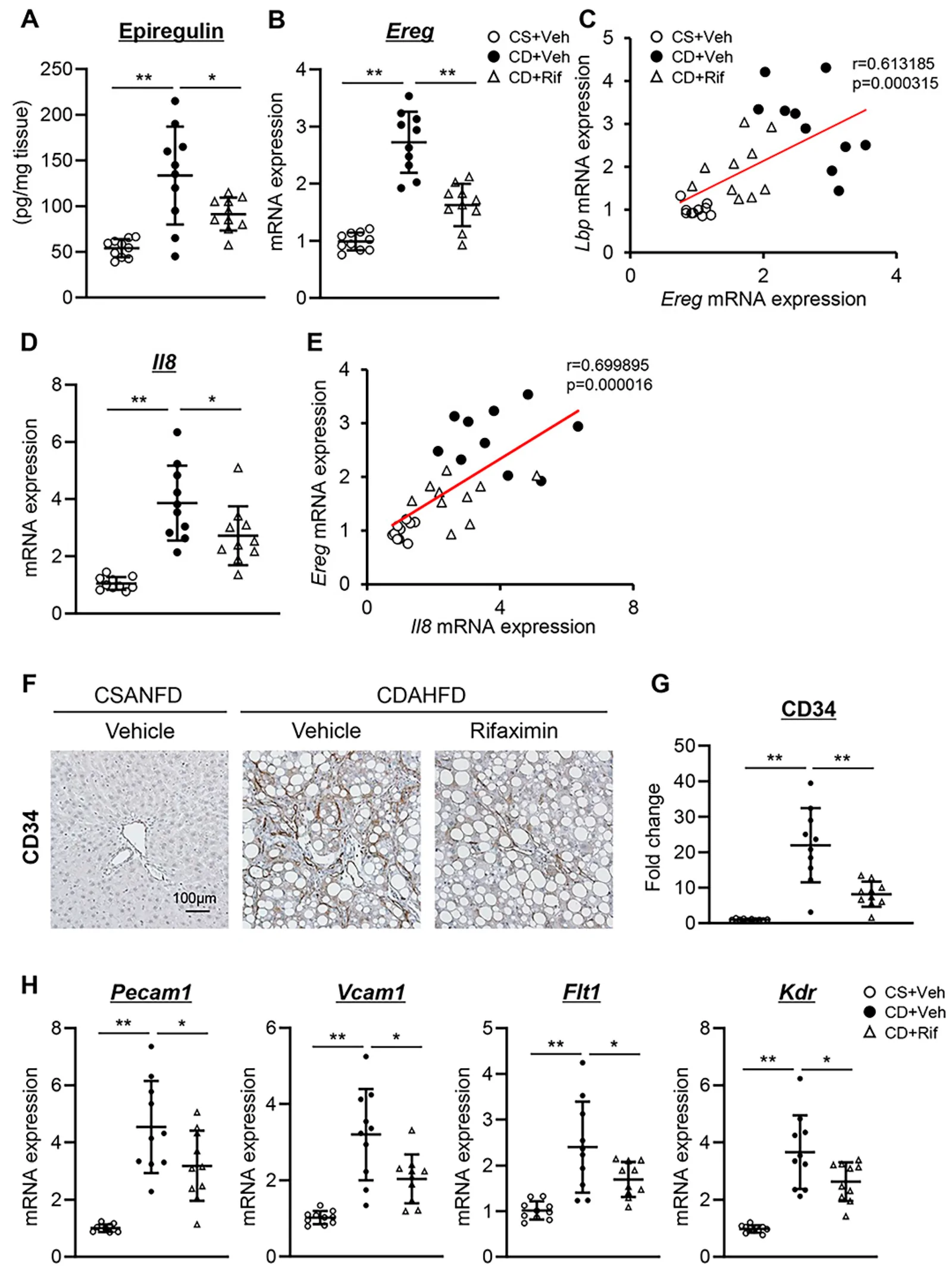
Effect of rifaximin on epiregulin and intrahepatic angiogenesis in CDAHFD-fed rats. (**A**,**B**) Hepatic expression of epiregulin at (**A**) protein and (**B**) mRNA levels. (**C**) Pearson’s correlation between *Ereg* and *Lbp* mRNA levels in all experimental rats. (**D**) Hepatic mRNA level of *IL-8*. (**E**) Pearson’s correlation between *IL-8* and *Ereg* mRNA levels in all experimental rats. (**F**) Representative microphotographs of CD34 staining of liver tissue. (**G**) Quantification of CD34-stained neovascularization in a high-power field. (**H**) Hepatic mRNA level of proangiogenic markers (*Pecam1*, *Vcam1*, *Flt1*, and *Kdr*). *Gapdh* was used as an internal control for qRT-PCR (**B**,**D**,**H**). Data are the mean ± SD (*n* = 10). Quantitative values are indicated as fold changes to the values of the CS+Veh group (**B**,**D**,**G**,**H**). * *p* < 0.05, ** *p* < 0.01, significant difference between groups determined by Student’s *t*-test.

Previous reports have demonstrated that epiregulin promoted HCC cell growth under the influence of LPS [26,27,28]. In line with the progression of hepatocarcinogenesis, hepatic epiregulin expression was increased at the protein and mRNA levels in CDAHFD-fed rats, which was ameliorated by rifaximin (Figure 4A,B). As shown in Figure 4C, the hepatic mRNA level of epiregulin (*Ereg*) was positively correlated with that of *Lbp* in the experimental rats (r = 0.613185, *p* < 0.001). Previous studies have demonstrated that the LPS-mediated upregulation of epiregulin contributes to tumor neovascularization via IL-8 signaling in HCC [26]. CDAHFD-fed rats had higher hepatic *IL-8* mRNA expression, which was reduced after treatment with rifaximin (Figure 4D). Notably, the hepatic mRNA levels of *Ereg* and *IL-8* demonstrated a positive correlation (r = 0.699895, *p* < 0.0001) (Figure 4E). Alongside this increased hepatic IL-8 expression, CD34-positive neovascularization was augmented in the liver of CDAHFD-fed rats, which was attenuated by treatment with rifaximin (Figure 4F,G). Consistently, treatment with rifaximin significantly reduced the hepatic mRNA levels of proangiogenic genes, specifically *Pecam1*, *Vcam1*, *Flt1*, and *Kdr* (Figure 4H).

### 2.5. Rifaximin Reinforced Intestinal Barrier Function in CDAHFD-Fed Rats

**Figure 5:**
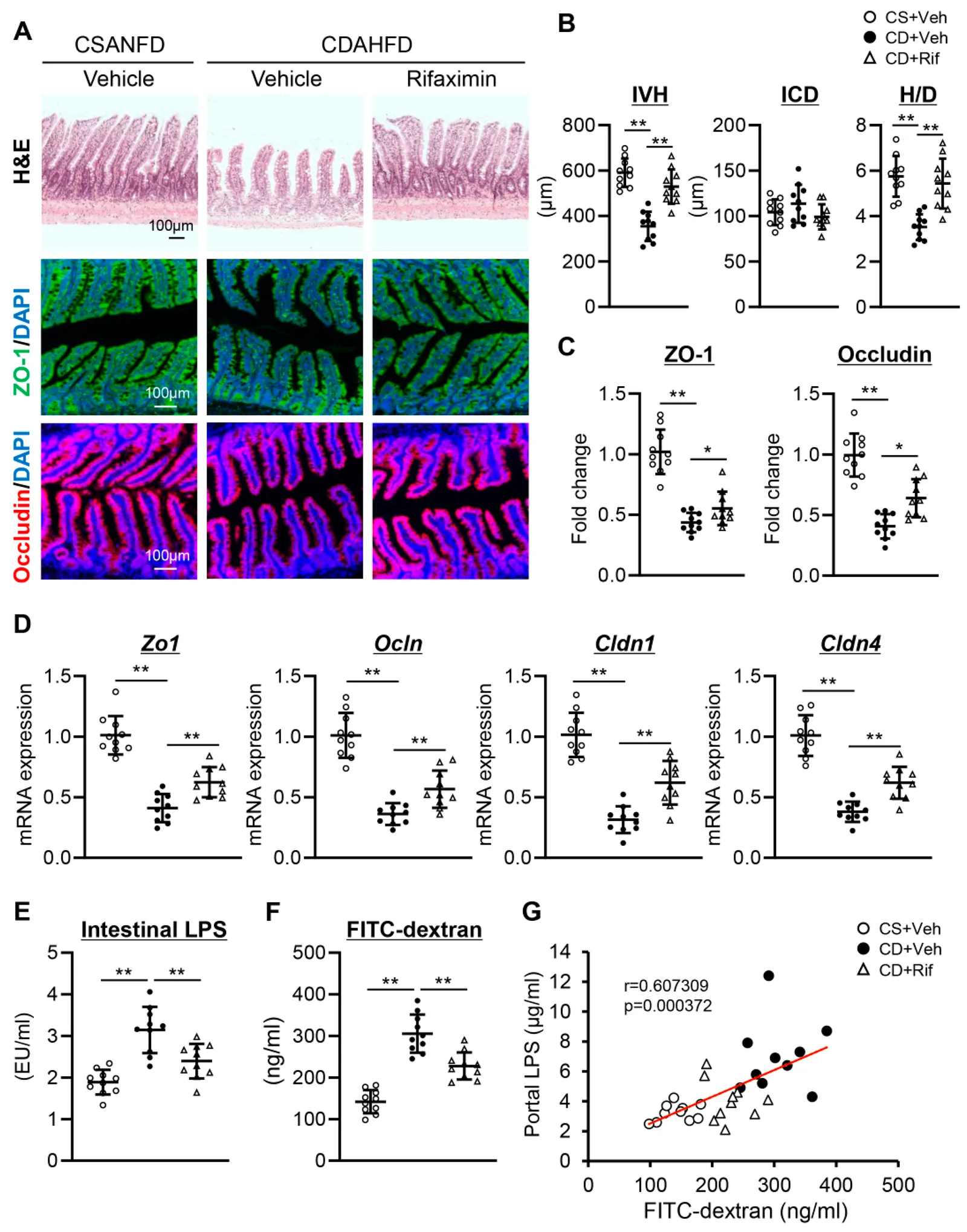
Effect of rifaximin on intestinal barrier function in CDAHFD-fed rats. (**A**) Representative microphotographs of H&E, ZO-1, and Occludin staining of colon tissue. Nuclei counterstained with DAPI. (**B**) Assessment of IVH, ICD, and H/D. (**C**) Semi-quantitation of ZO-1 and Occludin immuno-positive areas in high-power field. (**D**) Ileum mRNA level of tight junction proteins (*Zo1*, *Ocln*, *Cldn1*, and *Cldn4*). (**E**) Intestinal LPS level. (**F**) Portal levels of fluorescein isothiocyanate (FITC)-dextran (4 kDa). (**G**) Pearson’s correlation between the portal FITC-dextran and LPS level in all experimental rats. *Gapdh* was used as an internal control for qRT-PCR (**D**). Data are the mean ± SD (*n* = 10). Quantitative values are indicated as fold changes to the values of the CS+Veh group (**C**,**D**). * *p* < 0.05, ** *p* < 0.01, significant difference between groups determined by Student’s *t*-test.

The mechanism behind the reduction of portal LPS by rifaximin was investigated by analyzing its effect on intestinal barrier function. In CDAHFD-fed rats, a significant reduction in the IVH was observed, as well as a tendency toward an increase in the ICD, although the difference was not significant, and consequently, H/D was significantly reduced as compared to CSANFD-fed rats (Figure 5A,B). Notably, these atrophic changes were suppressed by treatment with rifaximin (Figure 5A,B). Immunofluorescent analysis revealed that the intestinal expression of ZO-1 and Occludin, which are markers of tight junction protein (TJP), was decreased in CDAHFD-fed rats and subsequently restored after rifaximin administration (Figure 5A,C). In addition, rifaximin also increased the intestinal mRNA expression of other TJP markers (*Cldn1* and *Cldn4*) (Figure 5D). Compared to CSANFD-fed rats, the intestinal LPS level was significantly elevated in the CDAHFD-fed rats, which was attenuated by treatment with rifaximin (Figure 5E). Consistently, the FITC-dextran content of the CDAHFD-fed rats clearly increased, indicating intestinal hyperpermeability (Figure 5F), and this was sharply lowered by rifaximin treatment (Figure 5D). The portal levels of LPS and FITC-dextran notably had a positive correlation as well (r = 0.607309, *p* < 0.001) (Figure 5G).

## 3. Discussion

**Figure 6:**
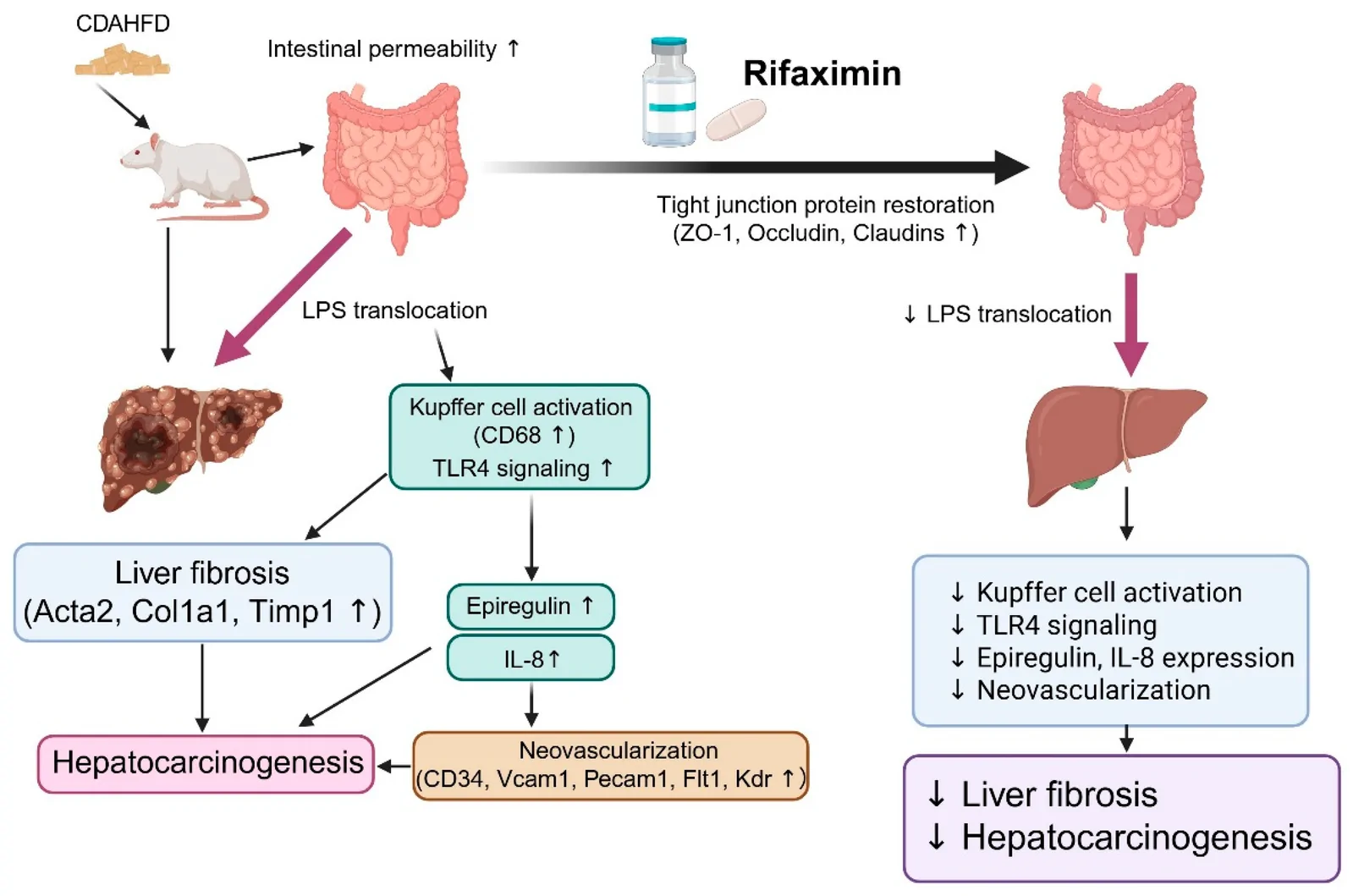
Graphic representation of the effect of rifaximin on MASH-related liver fibrosis and hepatocarcinogenesis.

Rifaximin, a nonabsorbable antibiotic, exhibited the protective effects against liver fibrosis and hepatocarcinogenesis in a CDAHFD-fed MASH rat model. These results highlight the gut–liver axis as a potential therapeutic target. In particular, modulating intestinal permeability and gut-derived LPS translocation was found to suppress inflammation-driven fibrogenesis and tumor progression in MASH (Figure 6). Our results also highlight a novel mechanism through which rifaximin attenuates hepatic angiogenesis by suppressing LPS-induced epiregulin and IL-8 signaling. One strength of this study is the use of the CDAHFD-fed rodent model, which faithfully recapitulates many key aspects of MASH in humans, including steatohepatitis, fibrosis, and early hepatocarcinogenesis [29,30,31]. This model enabled us to study the gut–liver axis under a relevant pathophysiological context and evaluate the effect of rifaximin across multiple disease stages [32].

A hallmark of MASH progression is persistent hepatic inflammation that orchestrates the fibrogenic and oncogenic processes. Our histological and biochemical analyses revealed that rifaximin mitigated the development of steatohepatitis, specifically by attenuating hepatocellular ballooning and inflammation, although steatosis remained unaffected. This selective action aligns with previous reports, which found that rifaximin does not significantly influence lipid accumulation in the liver but rather exerts anti-inflammatory effects by modulating TLR4 signaling [24,33]. This hepatoprotective role of rifaximin is further supported by the normalization of serum ALT levels and downregulation of hepatic proinflammatory cytokines. These anti-inflammatory effects were closely associated with reduced portal LPS concentrations and suppressed Kupffer cell activation as well. These findings highlight the central role of gut-derived endotoxins in sustaining hepatic inflammation in MASH.

A key finding of this study was the significant attenuation of liver fibrosis and profibrogenic gene expression after rifaximin administration. The fibrotic area was reduced by nearly 50%, and the hepatic hydroxyproline content, a surrogate for collagen deposition, markedly decreased. These findings are consistent with the notion that LPS-mediated TLR4 activation promotes HSC activation and fibrogenesis [18,19,34]. Rifaximin likely disrupts this pathological mechanism by reinforcing intestinal barrier integrity, thereby reducing LPS leakage into the portal circulation and dampening the downstream inflammatory cascade. Rifaximin also restored TJP expression (i.e., ZO-1, Occludin, claudin-1, and claudin-4) in the intestinal epithelium, underscoring its beneficial effect on gut barrier function [24,33,35]. This improvement in intestinal permeability was quantitatively supported by the reduced serum FITC-dextran levels and a strong correlation between portal LPS and FITC-dextran, suggesting the central role of reduced endotoxemia in ameliorating hepatic injury.

The most novel contribution of our study is that rifaximin suppressed early hepatocarcinogenesis in CDAHFD-fed rats by inhibiting angiogenesis-related pathways. Although rifaximin has been shown to attenuate hepatic inflammation and fibrosis, its effect on hepatocarcinogenesis—particularly by modulating tumor-promoting neovascularization—has not been previously elucidated [23,24,33]. In our study, rifaximin substantially inhibited neoplastic transformation, as evidenced by the significantly reduced number of GST-P^+^ PNLs and concurrent suppression of proliferation-related genes (i.e., *Mki67*, *Pcna*, *Ccnb1*, *Ccnb2*).

Epiregulin, a member of the epidermal growth factor family that binds to EGFR and ErbB4 receptors, was identified as a mechanistic link between LPS-TLR4 signaling and hepatocarcinogenesis [36,37]. It promotes cell proliferation, differentiation, and tissue repair, with roles in cancer progression, inflammation, and wound healing [36,37,38]. In a previous study, TLR4-dependent HCC promotion in the early phases of the disease is predominantly mediated by the TLR4-dependent secretion of epiregulin by HSCs [28]. Additionally, our recent study suggested that epiregulin facilitates cellular communication between HCC cells and activated HSCs, thereby promoting the progression of HCC by enhancing cancer cell proliferation, migration, invasion, and tumor angiogenesis caused by LPS stimulation [26]. In the present study, rifaximin downregulated epiregulin expression at both the mRNA and protein levels, with a strong positive correlation between hepatic *Lbp* and *Ereg* mRNA levels. Thus, through the suppression of LPS-TLR4 signaling, rifaximin indirectly mitigates epiregulin-driven oncogenic signaling.

Epiregulin enhances tumor neovascularization by inducing IL-8, a potent proangiogenic chemokine [26,39]. In our study, hepatic IL-8 expression was significantly reduced by rifaximin, with a positive correlation between *Ereg* and *IL-8* expression. Importantly, this molecular downregulation was reflected at the histological level, where CD34-positive neovascularization was markedly suppressed in CDAHFD-fed rats with rifaximin treatment. Furthermore, the reduction in the expression of key angiogenic genes (i.e., *Pecam1*, *Vcam1*, *Flt1*, and *Kdr*) indicates that rifaximin effectively inhibits the angiogenic switch, which is critical for tumor growth and metastasis. These findings are important, especially considering the strong association between angiogenesis and HCC progression. Increased microvessel density, vascular remodeling, and endothelial cell activation are all features of the tumor microenvironment in HCC [40,41]. Thus, by targeting the LPS-induced epiregulin–IL-8 axis, rifaximin prevents not only fibrotic progression but also the establishment of a protumorigenic vascular network.

Several limitations must be considered. First, while the suppression of GST-P^+^ PNLs suggests the chemopreventive effect of rifaximin, long-term studies are needed to confirm how it influences the incidence and onset of HCC. Second, although our results suggest the role of the LPS–epiregulin–IL-8 pathway in angiogenesis, the exact cellular sources and molecular intermediaries need to be further elucidated. Additional studies using cell-specific knockout models or inhibitors can help clarify these mechanisms. Third, our recent investigation employing the CDAA-induced MASH model demonstrated that rifaximin administration did not induce significant alterations in the composition of the gut microbiota [24,42]. Moreover, in vitro experiments utilizing human intestinal epithelial cells revealed that rifaximin exerts a direct protective effect on intestinal barrier integrity, primarily through activation of the pregnane X receptor (PXR) [24,42]. In this study, rifaximin also reduced intestinal LPS levels, suggesting that its effect may not be mediated by changes in the gut microbiota, based on these reported data. However, the finding that rifaximin does not affect the gut microbiota has only been observed in a specific MASH model, and verification using other models is considered necessary. Moreover, although the FITC-dextran assay was used to assess intestinal permeability, this method measures systemic absorption and does not provide information on which specific segments of the gut exhibit barrier disruption. Future studies employing segmental permeability assessments or imaging-based techniques would be necessary to localize the sites of intestinal barrier impairment. Finally, this study was conducted only in male rats, and therefore, potential sex-specific differences in disease progression or therapeutic response to rifaximin were not evaluated. Future studies incorporating both sexes are warranted to assess gender-related variations. Notably, rifaximin already has a well-established safety profile, with approval for use in patients with hepatic encephalopathy, making it easy to repurpose for MASH. This could provide an accessible and cost-effective strategy for preventing or halting the progression of HCC, particularly in patients with high LPS burden or intestinal barrier dysfunction. Additionally, LPS and epiregulin levels emerged as potential biomarkers for identifying patients who may benefit most from rifaximin therapy.

In conclusion, this study provides strong preclinical evidence that rifaximin can ameliorate the progression of MASH by reinforcing intestinal barrier function, reducing portal LPS translocation, suppressing Kupffer cell activation, and attenuating downstream fibrogenic, proliferative, and angiogenic pathways. Overall, these effects interrupt the gut–liver axis and the LPS–TLR4–epiregulin–IL-8 cascade. Therefore, rifaximin offers a promising strategy for the prevention of liver fibrosis and hepatocarcinogenesis in MASH. Further clinical investigations are warranted to validate these findings and explore the actual clinical impact of rifaximin.

## 4. Materials and Methods

### 4.1. Animal Experiments

All procedures were conducted over a 12-week period. Male Fischer 344 rats (*n* = 30, 6 weeks old, 130–150 g body weight; CLEA Japan, Tokyo, Japan) were housed under controlled conditions (temperature: 23 ± 3 °C; humidity: 50 ± 20%; 12-h light/dark cycle). The rats were randomly assigned to three groups (*n* = 10 per group) that received the following treatments: (1) a choline-supplemented L-amino acid-defined, normal-fat diet (CSANFD) (Research Diets Inc., New Brunswick, NJ, USA) with vehicle; (2) a choline-deficient, L-amino acid-defined, high-fat diet (CDAHFD) (Research Diets Inc.) with vehicle; and (3) a CDAHFD with oral rifaximin (30 mg/kg/day, dissolved in water) [43]. Rifaximin was generously provided by ASKA Pharmaceutical Co., Ltd. (Tokyo, Japan). Lactose hydrate (FUJIFILM Wako Pure Chemical Corporation, Osaka, Japan) was used as the vehicle in all the relevant groups. After 12 weeks, the rats were anesthetized, and laparotomy was performed to collect blood samples from the aorta and portal vein. The liver and ileum tissues were excised and promptly fixed in neutral-buffered formalin for histological evaluation. 

### 4.2. Histological Analyses

Rat liver and ileum tissues were harvested, fixed in 4% paraformaldehyde at 4 °C overnight, then embedded in paraffin. The paraffin-embedded liver specimens were processed into 5-μm sections and stained with hematoxylin and eosin and Sirius Red (performed at Narabyouri Research Co., Nara, Japan). The progression of steatohepatitis was analyzed using a non-alcoholic fatty liver disease activity score (NAS): severity of steatosis (0–3 points), lobular inflammation (0–2 points), and hepatocyte ballooning (0–3 points), originally developed for human NASH histology, without modification, as previously described [44]. NAS and scores for fibrosis (0–4 points) were determined as previously described by three experienced pathologists [44].

Intestinal villus height (IVH), intestinal crypt depth (ICD), and the ratio of villus height to crypt depth (H/D) were assessed using imaging software (ImageJ 64-bit Java 1.8.0, NIH, Bethesda, MD, USA) [45]. IVH is measured from the tip of the intestinal villus to the mouth of the corresponding intestinal crypt, while ICD is measured from the mouth of the intestinal crypt to its base [45].

### 4.3. Immunohistochemical and Immunofluorescence Analyses

For immunohistochemical analysis, the liver tissue sections were deparaffinized and rehydrated, then incubated with 10% hydrogen peroxide for 10 min to block endogenous peroxidase activity. Antigen retrieval was performed by heating the sections in 0.01 mol/L sodium citrate buffer for 30 min. The tissues were incubated in 2% bovine serum albumin for 1 h at room temperature to block non-specific binding. Afterwards, the sections were incubated overnight at 4 °C with the following primary antibodies: rabbit polyclonal anti-placental glutathione S-transferase (GST-P; 311, 1:300, Medical and Biological Laboratories, Nagoya, Japan), mouse monoclonal anti-CD68 (GTX41868, 1:50, GeneTex, Irvine, CA, USA), and rabbit monoclonal anti-CD34 (ab81289, 1:2500, Abcam, Cambridge, UK). The sections were washed with phosphate-buffered saline, then incubated with an appropriate secondary antibody at room temperature (20–25 °C) for 30 min. Immunoreactive signals were visualized using 3,3′-diaminobenzidine as the chromogen.

For immunofluorescence, Zonula occludens-1 (ZO-1; 61-7300, 1:100, Thermo Fisher Scientific, Waltham, MA, USA) and Occludin (71–1500, 1:100, Thermo Fisher Scientific) were applied to the ileum sections, using Alexa Fluor™ 488 and 594 (Thermo Fisher Scientific) as their respective secondary antibodies. All slides were captured, then 5 images at 20× magnification were randomly selected and analyzed with a computerized ImageJ 64-bit Java 1.8.0 (NIH).

### 4.4. Quantitative Real-Time PCR

The total RNA was extracted from rat liver and ileum tissues using the RNeasy Mini Kit (Qiagen, Hilden, Germany) as per the manufacturer’s protocol. A spectrophotometer was used to assess RNA purity and concentration. Complementary DNA (cDNA) was synthesized from 1 μg of total RNA using the High-Capacity RNA-to-cDNA™ Kit (Thermo Fisher Scientific). Quantitative real-time PCR was performed using SYBR™ Green PCR Master Mix (Thermo Fisher Scientific) on a StepOnePlus™ Real-Time PCR System (Thermo Fisher Scientific). Gene expression levels were normalized to the housekeeping gene glyceraldehyde-3-phosphate dehydrogenase (*Gapdh*), and relative expression levels were calculated using the comparative Ct method (2^−ΔΔCt^). All reactions were run in triplicate. The specificity of amplification was confirmed via melt curve analysis. The primer sequences are listed in Table S1.

### 4.5. Measurement of LPS Levels in Portal Vein and Intestinal Tissue

Bioactive LPS in the portal vein was determined via serial dilution and plating 24 h after infection using the HEK-Blue^TM^ LPS Detection Kit 2 (InvivoGen, San Diego, CA, USA) as per the manufacturer’s protocol. Intestinal LPS levels were determined using Rat LPS ELISA Kit (ELK biotechnology, Denver, CO, USA) according to the manufacturer’s protocol.

### 4.6. Hepatic Triglyceride (TG) Measurement

The TG content of the rat liver tissue was determined using the Triglyceride-Glo™ Assay (Promega, Madison, WI, USA) as per the manufacturer’s protocol.

### 4.7. Measurement of the Hepatic Epiregulin Level

The epiregulin level in rat liver tissue was measured using a Rat Epiregulin ELISA Kit (Novus Biologicals, Centennial, CO, USA) as per the manufacturer’s protocol.

### 4.8. Liver Hydroxyproline Content Measurement

The liver hydroxyproline content was determined using the Hydroxyproline Assay Kit (Abcam), as per the manufacturer’s protocol. Liver tissue (approximately 10 mg) was homogenized in distilled water and hydrolyzed with 100 μL of 12 N hydrochloric acid (HCl) at 120 °C for 3 h in a pressure-sealed vial. After hydrolysis, the samples were cooled to room temperature and centrifuged to remove particulate matter, then the supernatant was collected, neutralized, and treated with chloramine T solution at room temperature for 20 min to facilitate oxidation. Afterwards, the samples were incubated with Ehrlich’s reagent at 65 °C for 20 min. Absorbance was measured at 560 nm using a microplate reader. The hydroxyproline levels were quantified in comparison to a standard curve generated using known concentrations of hydroxyproline. The final values were normalized to the tissue weight, expressed as micrograms of hydroxyproline per milligram of tissue (μg/mg).

### 4.9. Gut Permeability Assay

Intestinal permeability was assessed using a 4-kDa fluorescein isothiocyanate (FITC)-dextran solution (Sigma–Aldrich, St. Louis, MO, USA) as previously described [46]. The rats were subject to 6 h of fasting, after which FITC-dextran was orally administered (40 mg/kg) in a volume of 200 μL, then portal vein blood samples were collected after 1 h. To determine intestinal permeability, the concentration of FITC-dextran in the plasma was measured via fluorescence spectroscopy using a NanoDrop 3300 fluorospectrometer (Thermo Fisher Scientific) (excitation wavelength: 490 nm, emission wavelength: 520 nm).

### 4.10. Statistical Analyses

Statistical analyses were performed using GraphPad Prism version 9.00 (GraphPad Software, La Jolla, CA, USA). Data was expressed as the mean ± standard deviation (SD) from a minimum of three independent experiments, and the error bars in all figures represent the SD. Differences between the two groups were assessed using Student’s *t*-test (parametric). Correlations among variables were determined using Pearson’s correlation coefficient. Statistical significance was set at *p* < 0.05.

## Data Availability

The data that support the findings of this study are available from the corresponding author upon reasonable request.

## Supplementary Materials

The following supporting information can be downloaded at: https://www.mdpi.com/article/10.3390/ijms26146710/s1.
