# Correction: Nishimura et al. Rifaximin Attenuates Liver Fibrosis and Hepatocarcinogenesis in a Rat MASH Model by Suppressing the Gut–Liver Axis and Epiregulin–IL-8-Associated Angiogenesis. *Int. J. Mol. Sci.* 2025, *26*, 6710

**DOI:** 10.3390/ijms27157041

**Published:** 2026-08-06

**Authors:** Naoki Nishimura, Kosuke Kaji, Norihisa Nishimura, Junichi Hanatani, Tatsuya Nakatani, Masafumi Oyama, Akihiko Shibamoto, Yuki Tsuji, Koh Kitagawa, Shinya Sato, Tadashi Namisaki, Satoru Tamaoki, Hitoshi Yoshiji

**Affiliations:** 1 Department of Gastroenterology, Nara Medical University, Kashihara 634-8521, Nara, Japan; k147051@naramed-u.ac.jp (N.N.); nishimuran@naramed-u.ac.jp (N.N.); k136081@naramed-u.ac.jp (J.H.); k182324@naramed-u.ac.jp (T.N.); k184066@naramed-u.ac.jp (M.O.); a-shibamoto@naramed-u.ac.jp (A.S.); tsujih@naramed-u.ac.jp (Y.T.); kitagawa@naramed-u.ac.jp (K.K.); shinyasato@naramed-u.ac.jp (S.S.); tadashin@naramed-u.ac.jp (T.N.); yoshijih@naramed-u.ac.jp (H.Y.); 2 Medical Affairs Department, ASKA Pharmaceutical Co., Ltd., Minato-ku 108-8532, Tokyo, Japan; tamaoki-s@aska-pharma.co.jp

## Figure Correction

In the original publication [1], there was a mistake in Figure 2 as published. Due to an inadvertent error during the data archiving process, a sample mismatch occurred, and incorrect representative images from a parallel study were included in Figure 2A. The corrected Figure 2A (representative pictures) and Figure 2B (quantitative analysis) appear below.

## Text Correction

There was an error in the original publication [1]. The Conflicts of Interest statement incorrectly stated that the study was funded by ASKA Pharmaceutical Co., Ltd. The corrected Conflicts of Interest Section appears bellow.

**Conflicts of Interest:** ASKA Pharmaceutical Co., Ltd. provided the active pharmaceutical ingredient Rifaximin. Hitoshi Yoshiji has received speaker honoraria from ASKA Pharmaceutical Co., Ltd.

The authors state that the scientific conclusions are unaffected. This correction was approved by the Academic Editor. The original publication has also been updated.

## Figures and Tables

**Figure 2:**
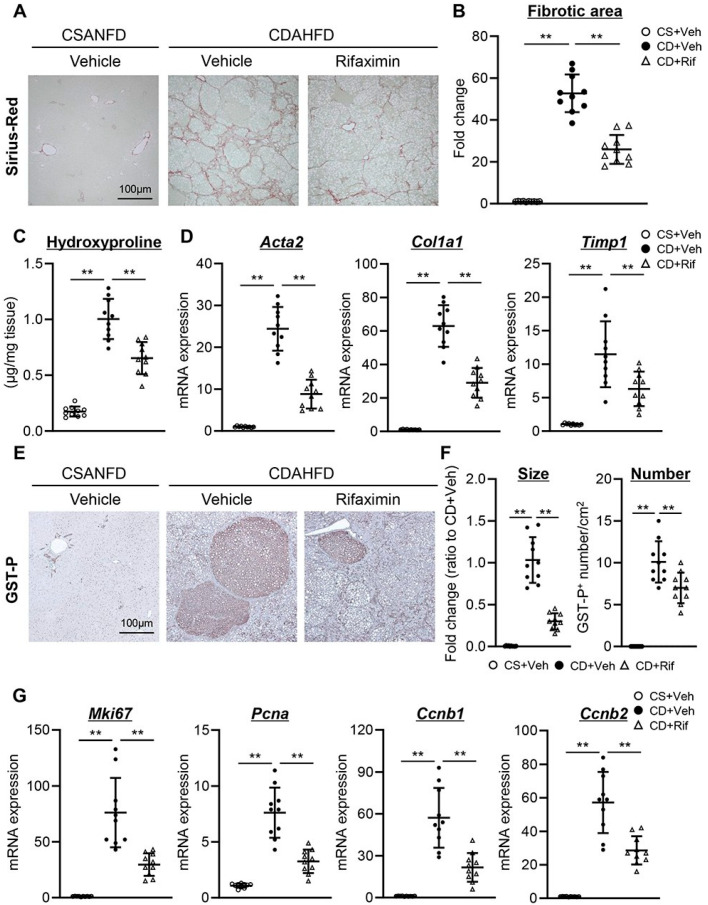
Effect of rifaximin on CDAHFD-induced liver fibrosis and hepatocarcinogenesis. (**A**) Representative microphotographs of Sirius-Red staining of liver tissue. (**B**) Quantification of Sirius-Red stained fibrotic area in high-power field. (**C**) Hepatic concentration of hydroxyproline. (**D**) Hepatic mRNA level of profibrotic markers (*Acta2*, *Col1a1*, and *Timp1*). (**E**) Representative microphotographs of placental glutathione transferase (GST-P)-positive preneoplastic foci in the experimental rats. (**F**) Number and relative size of GST-P-positive neoplastic lesions per square centimeter. (**G**) Hepatic mRNA level of genes related to cell proliferation and cell cycle (*Mki67*, *Pcna*, *Ccnb1*, and *Ccnb2*). *Gapdh* was used as an internal control for qRT-PCR (**D**,**G**). Data are the mean ± SD (n = 10). Quantitative values are indicated as fold changes to the values of the CS+Veh group (**B**,**D**,**G**) or the CD+Veh group (**F**). ** *p* < 0.01, significant difference between groups determined by Student’s *t*-test.
