# Supplementary material for: Rifaximin Attenuates Liver Fibrosis and Hepatocarcinogenesis in a Rat MASH Model by Suppressing the Gut–Liver Axis and Epiregulin–IL-8-Associated Angiogenesis
Source: Int J Mol Sci. 2025 Jul 12;26(14):6710. doi: 10.3390/ijms26146710 (PMC12294754; doi:10.3390/ijms26146710)
Supplement: Supplementary file 1 [file ijms-26-06710-s001.zip › ijms-3707022-supplementary.pdf]

Supplementary Table S1. List of primers used in q-PCR.

Gene	Sense (5'-3')	Antisense (5'-3')
Acta2	ACTGGGACGACATGGAAAAG	CATCTCCAGAGTCCAGCACA
Col1a1	TGCTGCCTTTTCTGTTCCTT	AAGGTGCTGGGTAGGGAAGT
Timp1	GCCTCTGGCATCCTCTTGTT	GTCGAATCCTTTGAGCATCTTAG
Mki67	ATTTCAGTTCCGCCAATCC	GGCTTCCGTCTTCATACCTAAA
Pcna	GGCGTGAACCTACAGAGCAT	CACAGGAGATCACCACAGCA
Ccnb1	ACAACGGTGAATGGACACCA	GCCACGGTTCACCATGACTA
Ccnb2	GACCGGCTCAAGTGGCTAAG	TCAGAG AAAGCTTGGCAGAGG
Lbp	AAGGCGCAAGTGAGACTGAT	AGTCGAGGTCGTGGAGCTTA
Cd14	CAACTTCTCAGATCCGCAGC	ACGCAGGGTTCCGAATAGAA
Tlr4	TGCTCAGGACATGGCAGTTTC	TCAAGGCTTTTCCATCCAAC
Tnfα	ACTCCCAGAAAAGCAAGCAA	CGAGCAGGAATGAGAAGAGG
Il1b	GCTGACAGACCCCAAAAGATT	ATCTGGACAGCCCAAGTCAA
Il6	CCGGAGAGGAGACTTCACAG	ACAGTGCATCATCGCTGCTGTTC
Ccl2	AGCCAACTCTCACTGAAGC	GTGAATGAGTAGCAGCAGGT
Ereg	ATCACAGTCGTCGGTTCCAC	AGGCACACTGTTATCCCTGC
Il8	CTGTAAGAGGGTTCCAATG	AGGTTCAGCACGTAGACAT
Pecam1	CCAGAAAGACAAGGCGATCG	CGGCTGGAGGAGAGTTCTAG
Vcam1	ACTGTGACCTGTCAGCGAAG	TTAGGGACCGTGCAGTTGAC
Flt1	GAATTATTTTAGGACCAGGA	AAACTCCCACTTGCTGGCAT
Kdr	AAGCAAATGCTCAGCAGGAT	TAGGCAGGGAGAGTCCAGAA
Zo1	ACCGGAAGAAGTTTCGAGAGC	CTGTACTGTGAGGGCAACGG
Ocln	GAGGGTACACAGACCCCAGA	CAGGATTGCGCTGACTATGA
Cldn1	AGGTCTGGCGACATTAGTGG	GAAGGTGTTGGCTTGGGATA
Cldn4	TTTTCGTGCTCCCCAACCTT	CGATGTTGCTGCCGATGAAG
Gapdh	AGCTGAACGGGAAGCTCACT	CATTGAGAGCAATGCCAGCC
